# Selective Dorsal Rhizotomy for the Treatment of Spastic Triplegic Cerebral Palsy

**DOI:** 10.7759/cureus.9204

**Published:** 2020-07-15

**Authors:** TS Park, Susan Joh, Deanna M Walter, Matthew B Dobbs

**Affiliations:** 1 Neurological Surgery, Washington University School of Medicine, St. Louis Children's Hospital, St. Louis, USA; 2 Pediatric Neurosurgery, Washington University School of Medicine, St. Louis Children's Hospital, St. Louis, USA; 3 Pediatric Orthopaedic Surgery, Washington University School of Medicine, St. Louis Children's Hospital, St. Louis, USA

**Keywords:** spastic triplegia, cerebral palsy, selective dorsal rhizotomy, ambulation, spasticity

## Abstract

Background

Spastic triplegia is a recognized subtype of cerebral palsy (CP). In the course of treating spastic triplegic children with selective dorsal rhizotomy (SDR), we found that some children who had “minimal or mild involvement” in the stronger arm improved strikingly after undergoing SDR. Some of them became independent ambulators, which was an outcome that is not usually achieved in spastic quadriplegic children. However, the literature currently contains no data on the natural clinical course and the effects of CP interventions on spastic triplegia.

Objectives

Our aim was to elucidate the clinical characteristics of spastic triplegia and the effects of SDR on functional outcomes and the quality of life after childhood SDR.

Methods

The Institutional Review Board of the Washington University School of Medicine approved this quality of life survey (number: 201908177). The subjects of this study were children and adults (ages: 3.9-23.8 years at the time of the survey completion; mean: 12.1 ± 5.1 years) with spastic triplegic CP who had undergone SDR (ages: 2.2-15.9 years; mean: 6.1 ± 3.2 years) between 2003 and 2018 at the St. Louis Children’s Hospital. The follow-up period ranged from 1-16 years (mean: 6.0 ± 4.3 years). The study included a 76-patient cohort selected from a total of 253 spastic triplegic CP patients who had undergone SDR. All 253 patients were contacted via email or postal mail soliciting their participation in the study including the survey. The cohort included all patients who responded. The survey included questions on demographic information, quality of life, perceptions of health and the SDR procedure, motor and ambulatory functions, braces and orthotics, pain issues, side effects of SDR, and post-SDR treatment.

Results

Thirteen patients had presented with scissored gait, and these patients had undergone partial ventral rhizotomy (PVR) of L1-3 ventral roots immediately after the completion of SDR. Of note, 91% of 76 patients reported that SDR improved their quality of life, and 93% would recommend the procedure to other patients. After SDR, 21 more patients were able to run, 14 more played recreational sports, and 18 more could walk without using walking aids. Sixteen fewer patients used a wheelchair for long-distance walking and in crowds; 37 and 32 patients reported an improvement in the more affected arm and hand, respectively. Sixty-eight patients were able to regularly strengthened their muscles at least once a week, and 60 patients regularly stretched their legs. However, 53 patients required assistance with bathing or showering, 50 with getting dressed, and 56 with grooming or hygiene. Forty-eight patients had orthopedic surgery after SDR. Percutaneous hamstring-lengthening was the most common type of orthopedic surgery performed. Three of 13 patients who received PVR and SDR required adductor release. Six patients used medications for spasticity or dystonia. No late side effects of SDR were observed.

Conclusions

Our report elucidates the clinical features of spastic triplegia before and after SDR. A distinct clinical feature was the wide variation in ambulatory functions, ranging from total independent walking to wheelchair mobility. The vast majority of patients felt that SDR improved their motor functions and quality of life. PVR also resulted in favorable outcomes, with only three of 13 patients requiring additional adductor release surgery. There were no late complications related to SDR surgery.

## Introduction

Spastic triplegia is a recognized subtype of spastic cerebral palsy (CP) [1]. The current consensus on spastic triplegia is that spastic weakness is found in both lower extremities and one upper extremity. It is often difficult to differentiate it from spastic quadriplegia as there is no clear definition for the latter condition. In our experience, the stronger arm is almost always affected to a varying degree in the patients, and we use “minimal to mild” involvement of the stronger arm as a diagnostic criterion of spastic triplegia. It is possible that some clinicians may classify some of our patients as cases of spastic quadriplegia.

We have observed that none of the spastic quadriplegic patients become independent ambulators after undergoing selective dorsal rhizotomy (SDR). The quadriplegic patients need a wheelchair or an assistive device to walk even after undergoing SDR. By contrast, children with spastic triplegia who have “minimal or mild involvement” of the stronger arm show markedly improved motor functions after SDR. Some of them even progress to walk independently. The current literature includes no data on the natural course of spastic triplegia and outcomes after any CP therapeutic intervention. In the present study, we examined the outcome of spastic triplegia patients in the areas of gross motor functions, arm improvement, living status, improvement of daily life activities, perceptions of SDR by parents and patients, and orthopedic surgery and other treatments after SDR.

## Materials and methods

The Institutional Review Board of the Washington University School of Medicine approved this quality of life survey (No. 201908177). We obtained consent from patients directly or from their guardians. The subjects of this study were children and adults (ages: 3.9-23.8 years; mean: 12.1 ± 5.1 years at the time of the survey completion) with spastic triplegic CP who had undergone SDR (ages: 2.2-15.9 years; mean: 6.1 ± 3.2 years) between 2003 and 2018 at the St. Louis Children’s Hospital. We gathered contact information from emails, mailing addresses, and phone numbers recorded in our clinic’s database and medical records. The study survey was sent out to potential participants electronically via email or physically via postal mail to soliciting their participation.

The survey included questions on demographic information, quality of life, perception of health, the SDR procedure, motor and ambulatory functions, braces and orthotics, pain issues, side effects of SDR, and post-SDR treatment. Demographic information included the date of birth, current living situation, student status, and employment. Questions about bladder function, loss of sensation, and back pain were included to assess the side effects of SDR. Participants who indicated a sensory loss in the lower limbs were asked to indicate the location of the numbness. Patients were asked the frequency, level (on a scale of 1-10), and location of the body pain. Participants were also inquired about scoliosis, post-SDR orthopedic interventions, and medications.

A five-point scale from poor to excellent was utilized to evaluate the perception of one’s personal health, as seen in Question 1 of the SF-36 health survey [2]. Yes/no/unsure questions were asked to understand whether the participants thought they had benefited from SDR and if they would recommend the procedure to other hemiplegic patients. They were also given the option to add any comments about their perception of SDR. Yes/no questions were used to evaluate the quality of life at the time of the survey. The queries included those related to receiving help with transferring positions, strengthening muscles, and stretching legs. Questions were asked about the patients’ independence since SDR, and engagement in recreational sports.

Patients were given the option to answer “Yes” or “No” to questions about motor function, including running, walking, and sitting. These questions were based on criteria determining the Gross Motor Function Classification (GMFCS) level of the patient [3]. Regarding arm and hand function, patients were asked about the change in the movement of the affected arm and hand.

## Results

Study cohort

Initially, our target population consisted of 253 spastic triplegic CP patients who had undergone SDR. The definition of spastic triplegia covered the involvement of both lower limbs and one upper limb. The other upper limb was understood to be minimally or mildly involved. The research team obtained the contact information of the patients from the database of the Center for Cerebral Palsy Spasticity at the St. Louis Children’s Hospital. Thirty-seven patients did not have updated emails or postal addresses and could not be contacted, leaving 216 potential participants. Seventy-eight patients responded. Two of the 78 patients who responded declined to participate. Thus, we eventually had a 76-patient cohort for our study, accounting for 35% of the initially considered participants.

All 76 patients had undergone the SDR procedure as described previously [4]. The age of patients at the time of surgery ranged between 2.2-15.9 years (mean: 6.1 ± 3.2 years). The age of patients at the time of completing the survey was 3.9-23.8 years (mean: 12.1 ± 5.1 years). The follow-up period ranged from 1.1 to 16.3 years (mean: 6.0 ± 4.3 years); 62% of the patients were male, and 38% were female. Thirteen patients had presented with scissored gait and these patients had undergone partial ventral rhizotomy (PVR) of L1-3 ventral roots immediately after the completion of SDR.

Gross motor functions and daily physical activities

Fifty-eight patients still required assistance in daily activities even after SDR. The patients required the most assistance with activities related to grooming and hygiene; 53 patients required assistance with bathing or showering, 50 with getting dressed, and 56 with grooming or hygiene. However, only 21 patients required help with eating (Table 1). Patients also reported daily physical activities: 68 patients regularly strengthened their muscles at least once a week, and 60 regularly stretched their legs. Thirty-one patients engaged in recreational sports (Table 2).

Despite a high proportion of patients still requiring assistance with daily living, many patients reported improvement in ambulation: 30 patients were able to run after SDR, whereas only nine patients had been able to run preoperatively (a 27% difference). Fourteen more patients enjoyed recreational sports, 21 more could run and jump, and 18 more could walk without using walking aids. Patients requiring extra body or trunk support to improve arm and hand function went down from 58% (44 patients) to 18% (14 patients) after SDR. Fewer patients relied on wheelchairs for long distances and crowds (down from 61 patients to 45 patients). However, there was no change in the number of patients (11) who relied on powered wheelchairs for self-mobility (Table 2).

**Table 1:** Daily life activities after SDR for 76 spastic triplegic patients SDR: selective dorsal rhizotomy

Activities of daily living	Number of patients (%)			
Assistance with daily living				
		Yes	No	
Help with eating		21 (28%)	54 (71%)	
Help with bathing or showering		53 (70%)	23 (30%)	
Help with using the toilet		39 (51%)	36 (47%)	
Help with getting dressed		50 (66%)	26 (34%)	
Help with grooming or hygiene		56 (74%)	20 (26%)	
Help with transferring from one position to another		26 (34%)	50 (66%)	
Daily exercise				
Regularly strengthen muscles		68 (89%)	8 (11%)	
Regularly stretch legs		60 (79%)	16 (21%)	
Play recreational sports		31 (41%)	45 (59%)	

**Table 2:** Gross motor functions before and after SDR in 76 spastic triplegic patients *Some patients required aid in more than one activity SDR: selective dorsal rhizotomy

Gross motor function*	Pre-SDR, number of patients (%)	Post-SDR, number of patients (%)
Able to run	9 (12%)	30 (39%)
Play any recreational sports	14 (18%)	28 (37%)
Able to run and jump	7 (9%)	28 (37%)
Able to walk without using walking aids	23 (30%)	41 (54%)
Able to use stairs without a handrail	3 (4%)	15 (20%)
Able to walk on any surface and environment	7 (9%)	27 (36%)
Able to walk using a walking aid (need to use a handrail for stairs)	31 (41%)	30 (39%)
Difficulty walking on uneven surfaces, slopes, or in crowds	63 (83%)	48 (63%)
Use of wheelchair for long distances or in crowds	61 (80%)	45 (59%)
Able to sit on one's own (cannot stand or walk without support)	55 (72%)	43 (57%)
Reliance on a wheelchair (home, school, and community)	38 (50%)	19 (25%)
Require extra body or trunk support to improve arm and hand function	44 (58%)	14 (18%)
Reliance on a powered wheelchair for self-mobility	11 (14%)	11 (14%)
Difficulty sitting on one's own and controlling head and body posture	17 (22%)	7 (9%)
Difficulty controlling own movements	25 (33%)	12 (16%)
Require special supportive chair	34 (45%)	16 (21%)
Lifted or hoisted by another person to move	27 (36%)	15 (20%)

Postoperative arm and hand improvement

Thirty-seven patients reported an improvement in the movement of the affected arm after SDR, but 36 saw no change, and one reported the condition of the arm to be worse after surgery. Two patients did not respond to questions about the movement of the affected arm, and one patient did not answer the question about the affected hand. Thirty-two patients reported improvement of the affected hand. No patient reported a worse outcome related to the movement of the affected hand (Table 3).

**Table 3:** Postoperative arm and hand function in 76 spastic triplegic patients *Some patients did not respond

Arm and hand function	Number of patients (%)		
	Better	Worse	Unchanged
Movement of affected arm*	37 (49%)	1 (1%)	36 (47%)
Movement of affected hand	32 (42%)	0 (0%)	43 (57%)

Patients' perception of SDR

Sixt-nine patients (91%) believed that SDR had improved their quality of life, and 71 patients (93%) said they would recommend the procedure to help other patients (Table 4).

**Table 4:** Perception of SDR in 76 spastic triplegic patients SDR: selective dorsal rhizotomy

Perception of SDR	Number of patients (%)		
	Yes	No	Unsure
SDR improved quality of life	69 (91%)	3 (4%)	4 (5%)
Would recommend SDR to others	71 (93%)	0 (0%)	5 (7%)

Postoperative pain

Thirty-seven patients reported having body pain post-SDR. Two patients reported constant pain, 45 occasional pain, and 36 reported no pain. Three patients did not have a response to body pain frequency. On a numerical scale of 1-10, with 1 being almost no pain and 10 being the worst pain imaginable, patients' responses ranged from 1 to 9 (mean: 3.3 ± 1.8). One patient answered with 9 and reported pain only in the ankle and foot. Others who reported a pain level of 5 and higher mostly reported pain in the general leg area and back. One patient reported a complete loss of sensation in any part of the legs. Two patients reported experiencing back pain directly related to the SDR. Four patients received treatment from a physician for their back pain, and no patient reported taking medication or seeking surgical treatment for back pain (Table 5).

**Table 5:** Post-SDR pain in 76 spastic triplegic patients *Some patients did not respond SDR: selective dorsal rhizotomy

Body pain	Number of patients (%)		
	Constant	Occasional	No pain
Frequency of body pain*	2 (3%)	35 (46%)	36 (47%)
	Numerical rating scale (0-10)		
Average pain score	Range: 1–9 (mean: 3.4 ± 1.8)		
Location of pain			
Knee	16 (21%)		
Ankle and foot	16 (21%)		
Back	16 (21%)		
Arm	2 (3%)		
Leg	22 (29%)		
Complete loss of sensation in any part of legs	1 (1%)		
Received treatment from physicians for back pain	4 (5%)		
Medications for back pain	0 (0%)		
Surgical treatment for back pain	0 (0%)		

Post-SDR orthopedic surgery and medication

Of note, 63% of 76 patients received percutaneous tendon-lengthening surgery. Surgery on hamstrings was the most common tendon- lengthening surgery. Six patients used medications for spasticity or dystonia (Table 6).

**Table 6:** Postoperative treatments in 76 spastic triplegic patients SDR: selective dorsal rhizotomy

Post-SDR treatment	Number of patients (%)
Tendon-lengthening surgery	48 (63%)
	Number and % of 48 tendon-lengthening patients
Hamstrings	30 (63%)
Achilles tendon	24 (50%)
Gastrocnemius	6 (13%)
Adductors	11 (23%)
Hip	13 (27%)
Medications for spasticity or dystonia	6 (8%)

Postoperative orthotics use

Most patients currently use braces or orthotics (59 patients). A high proportion of patients use ankle-foot orthosis (AFOs, 40 patients). Others use supramalleolar orthosis (SMOs, 15 patients), shoe inserts (10 patients), and others (four patients). Other braces and orthotics used include, but are not limited to, night orthotics, Bioness (Bioness Inc., Santa Clarita, CA), and immobilizers (Table 7).

**Table 7:** Postoperative use of orthotics in 76 spastic triplegic patients SMO: supramalleolar orthosis; AFO: ankle-foot orthosis

Use of braces or orthotics	Number of patients (%)
Currently using braces or orthotics	59 (78%)
Shoe inserts	10 (13%)
SMO	15 (20%)
AFO	40 (53%)
Other	4 (5%)

Perceptions of health 

Almost all patients (74 patients) reported that their health was good or better. Twenty-one patients reported excellent health, 34 very good, 19 good, two fair, and none reported poor health (Table 8).

**Table 8:** Postoperative perception of health in 76 spastic triplegic patients

Perception of health	Number of patients (%)
Excellent	21 (28%)
Very good	34 (45%)
Good	19 (25%)
Fair	2 (3%)
Poor	0 (0%)

Living situation, education, and employment

Sixty-seven patients reported living with their parents, six with a significant other, two with a roommate, and one reported living alone. Sixty-one patients were full-time students, and 10 were not students. Thirteen patients were employed at the time of the survey; two of the employed patients were working full-time, 10 were working part-time, and one patient did not specify the employment status (Table 9).

**Table 9:** Living situation and education and employment status of 76 spastic triplegic patients *Some patients did not specify

Living situation	Number of patients (%)
With significant other	6 (8%)
With parents	67 (88%)
With roommate	2 (3%)
Alone	1 (1%)
Education	
Full-time student	61 (80%)
Part-time student	5 (7%)
Not a student	10 (13%)
Employment	
Employed	13 (17%)
	(Number and % of employed patients*)
Full-time	2 (15%)
Part-time	10 (77%)

## Discussion

The present study was a retrospective analysis of survey responses from 76 patients out of 216 (35%) potential participants. Thus the study has some limitations [5,6]; however, we believe it still provides information that helps to understand the effects of SDR on motor functions, patients' perception of SDR, and the status of the daily life activities specific to a series of spastic triplegic patients. Our study is the first of its kind to discuss spastic triplegic patients. The significant findings of our study include: 1) the overall clinical characteristics of spastic triplegia are distinctly different from those of spastic quadriplegia; 2) the level of walking ability varies widely, and SDR improved walking ability in spastic triplegic patients; 3) SDR improved arm and hand function; 4) SDR upgraded various daily-life activities; 5) the patients' perception of SDR was overwhelmingly positive; 6) scissored gait is common in spastic triplegia, and PVR is an alternative to adductor release; 7) minimally invasive orthopedic surgery following SDR could replace the extensive multilevel orthopedic surgery.

It is remarkable that in our series of spastic triplegic patients, the preoperative walking ability varied from complete independent walking and playing recreational sports to a total reliance on wheelchairs. By contrast, none of our spastic quadriplegic patients could walk independently after SDR. Elaboration on a few clinical insights is worthwhile. First, after SDR, virtually all mobility-related activities and the quality of walking improved (Table 1). For patients who could walk independently before surgery, the ability to walk and dorsiflex the foot voluntarily on both sides-with better results on the less affected side-further improved after surgery. The presence of voluntary foot movement is a good indicator of the potential to become an independent walker after SDR. Second, some children under five years of age walked with the aid of walkers before surgery, and they can become independent walkers shortly after SDR. This particular group of children has voluntary foot movement even on the weaker side [7]. Third, in children older than seven years, SDR may improve only a single GMFCS level in general. If a child under the age of seven years walks with a walker before surgery, their walking outcome after surgery depends on the degree of arm and hand improvement after SDR. If one arm and hand are too weak to use crutches after SDR, crutch-walking is not an option. Unless they become independent walkers, they will rely on a walker and are less likely to use a single cane. It is significant that SDR improves the arm and hand functions and increases the chance of independent mobility. Fourth, besides mobility improvement, 41% of patients also gained the ability to play recreational sports, and the vast majority of patients were able to actively strengthen their muscles and stretch the lower limbs after SDR. Participation in recreational sports and exercise becomes easier after the reduction of spasticity.

In young children under five years of age, the scissoring itself prevents progression in gait development, and children cannot learn to walk. SDR alone cannot resolve scissoring caused by adductor contractures. We treated the scissored gait with PVR. The surgery entails dividing 30-50% of L1-L3 ventral roots at the time of SDR. Electrical stimulation of the ventral roots is not useful because of the overlapped innervation of the muscles in the lower limb. The level of ventral roots is identified by anatomy and muscle contraction in response to direct tap on the ventral root [8,9]. In our patients, PVR treated scissored gait while avoiding permanent wide-based gait, a well-known complication of the adductor release.

Thirteen patients (17%) had presented with scissored gait before the operation and all of them had gone on to receive PVR after SDR. PVR was successful in 10 of the 13 patients, and no adductor release was necessary after the procedure. The remaining three patients had recurrent scissoring and required percutaneous adductor lengthening. PVR is our first choice for the treatment of scissored gait due to the adductor contracture. They stretch the adductors daily while the adductor weakness after PVR improves for four months. In our experience, PVR seems more successful in children between the ages of two to age than in older children.

Significantly, 48 patients required orthopedic surgery after SDR. The correct orthopedic surgery following SDR complements SDR [10]. Orthopedic surgery should be timely and minimally invasive. Significantly, none of the patients underwent extensive multilevel bone surgery. The most common orthopedic procedures were percutaneous Achilles tendon repair and hamstring-lengthenings. Bony hip procedures were performed for 17% for hip subluxation. Concerning medical treatment, only 8% of our patients required medications for the treatment of spasticity or dystonia after SDR.

It is gratifying to note that that 91% (69 of 76 triplegic patients) of survey participants felt that SDR had improved their quality of life, and 93% (71 patients) said they would recommend SDR to other patients. None of the patients who did not participate in the survey reported that SDR was a total failure in all aspects of motor movements, daily life activities, independence, and confidence. It is encouraging that 74 of 76 patients reported good or better health even though some of them reported mild body pain. Another encouraging fact was that 80% (61 patients) of the participants were full-time students.

## Conclusions

Our study elucidated and analyzed the clinical features of spastic triplegia. A distinct clinical feature of spastic triplegia is the wide variation in ambulatory functions, ranging from total independent walking to wheelchair mobility. The vast majority of our patients felt that SDR had improved their motor functions and quality of life. The outcomes of PVR procedures were also favorable. There were no late complications related to SDR surgery.
